# Supplementary material for: Cell-to-Cell Transmission of HIV-1 and HIV-2 from Infected Macrophages and Dendritic Cells to CD4+ T Lymphocytes
Source: Viruses. 2023 Apr 22;15(5):1030. doi: 10.3390/v15051030 (PMC10222233; doi:10.3390/v15051030)
Supplement: Supplementary file 1 [file viruses-15-01030-s001.zip › viruses-2324313-supplementary.pdf]

Supplementary material

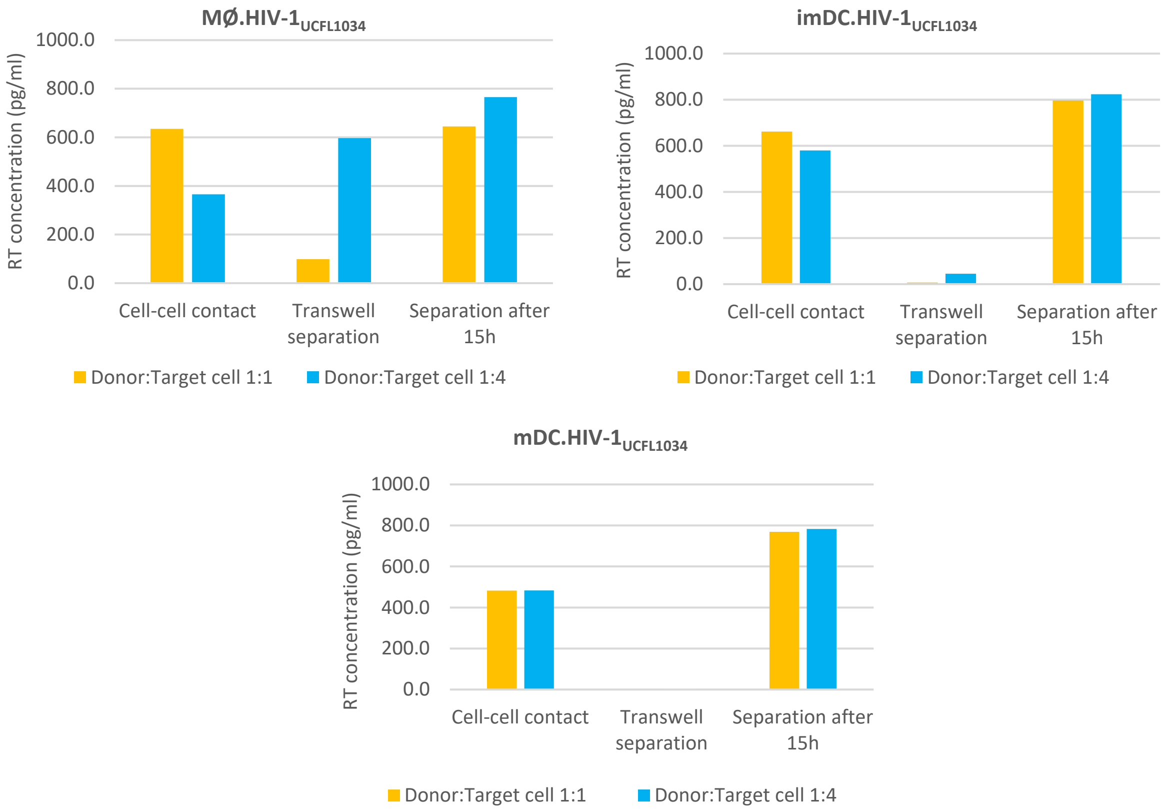

Figure S1. CD4+ T lymphocytes trans-infection by Mø, imDCs, and mDCs infected by HIV-1$_{UCFL1034}$ primary isolate. These experiments were performed in three conditions: i) cell-cell contact, allowing donor and target cells to be in coculture throughout the experiment; ii) donor and target cells separated by a transwell to prevent cell-cell contact; and iii) donor and target cells separated 15 hours after coculture. Peak RT concentration was measured in culture supernatants during a 7-day period after virus inoculation.

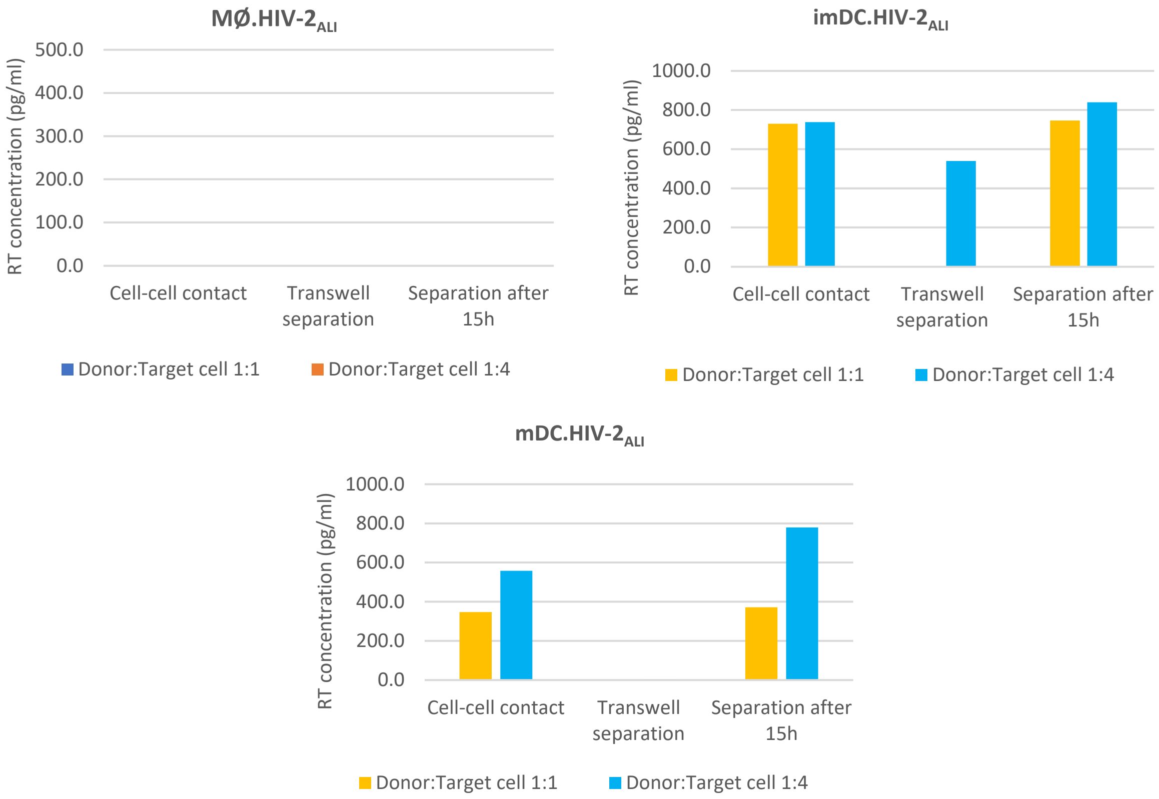

Figure S2. CD4+ T lymphocytes trans-infection by Mø, imDCs, and mDCs infected by HIV-1ALI primary isolate. These experiments were performed in three conditions: i) cell-cell contact, allowing donor and target cells to be in coculture throughout the experiment; ii) donor and target cells separated by a transwell to prevent cell-cell contact; and iii) donor and target cells separated 15 hours after coculture. Peak RT concentration was measured in culture supernatants during a 7-day period after virus inoculation.

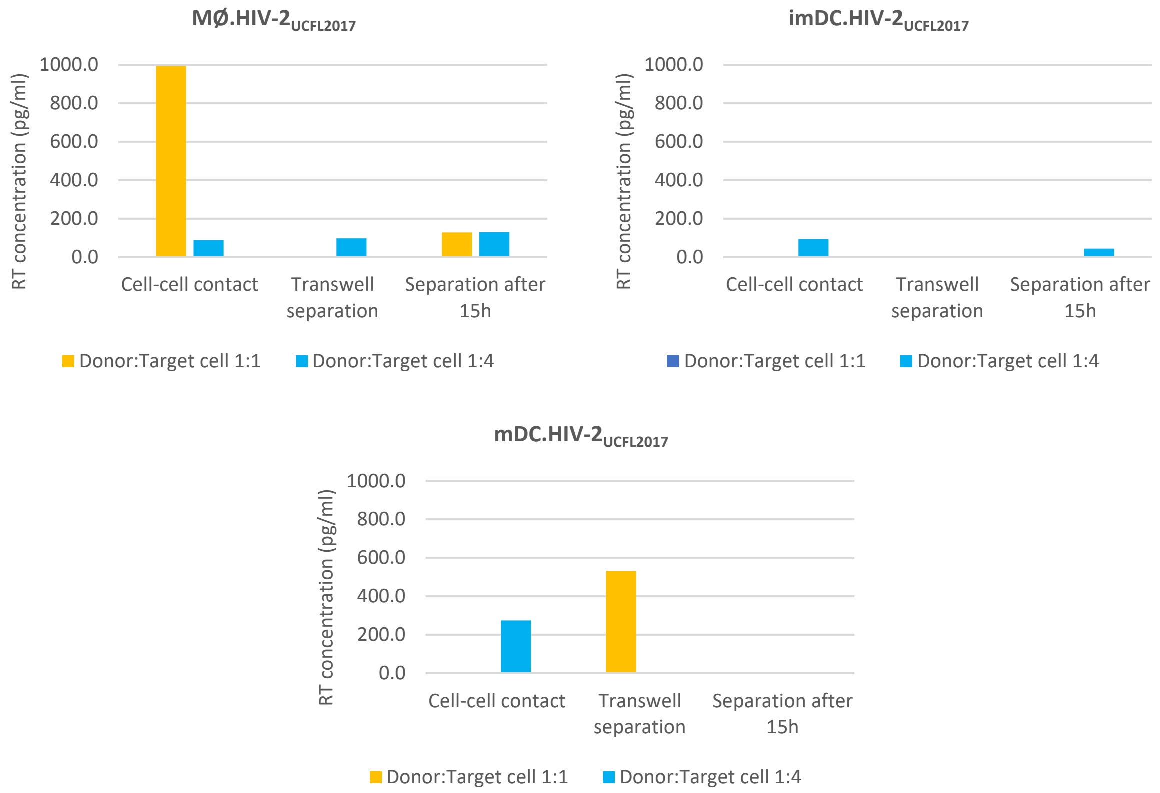

Figure S3. CD4+ T lymphocytes trans-infection by Mø, imDCs, and mDCs infected by HIV-1$_{UCFL2017}$ primary isolate. These experiments were performed in three conditions: i) cell-cell contact, allowing donor and target cells to be in coculture throughout the experiment; ii) donor and target cells separated by a transwell to prevent cell-cell contact; and iii) donor and target cells separated 15 hours after coculture. Peak RT concentration was measured in culture supernatants during a 7-day period after virus inoculation.

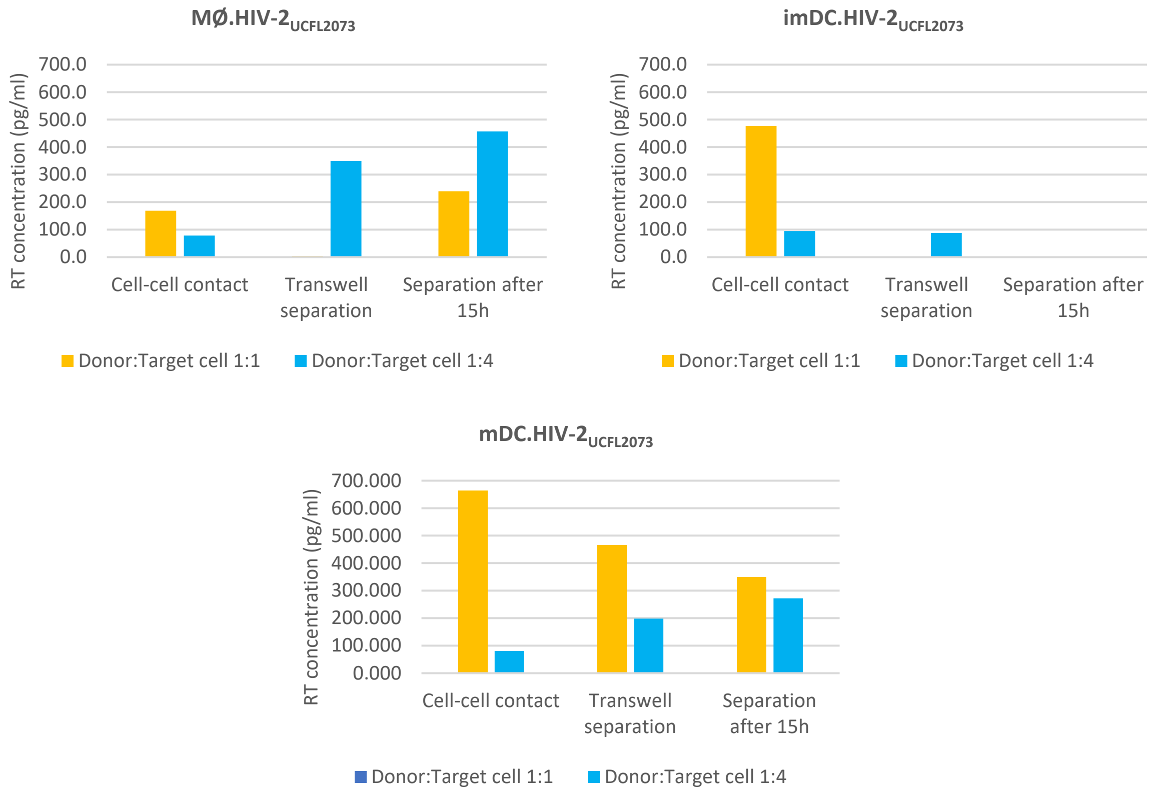

Figure S4. CD4+ T lymphocytes trans-infection by Mø, imDCs, and mDCs infected by HIV-1$_{UCFL2073}$ primary isolate. These experiments were performed in three conditions: i) cell-cell contact, allowing donor and target cells to be in coculture throughout the experiment; ii) donor and target cells separated by a transwell to prevent cell-cell contact; and iii) donor and target cells separated 15 hours after coculture. Peak RT concentration was measured in culture supernatants during a 7-day period after virus inoculation.

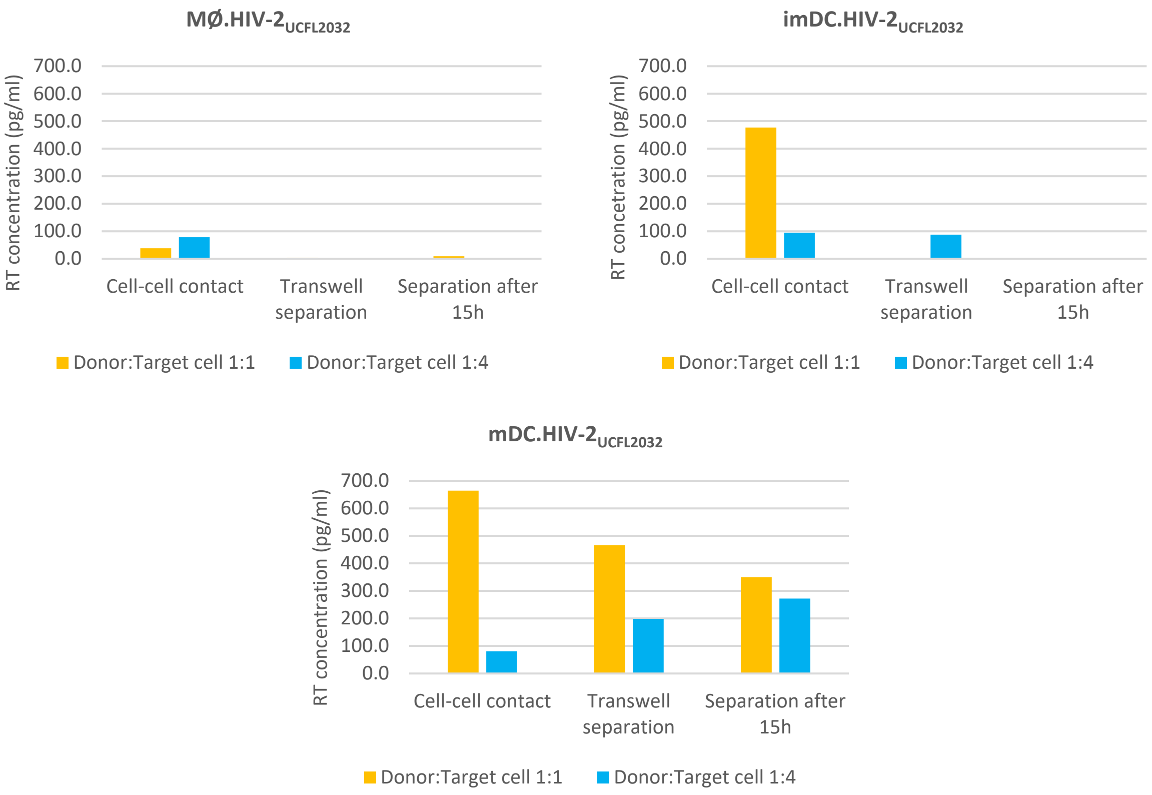

Figure S5. CD4+ T lymphocytes trans-infection by Mø, imDCs, and mDCs infected by HIV-1$_{UCFL2032}$ primary isolate. These experiments were performed in three conditions: i) cell-cell contact, allowing donor and target cells to be in coculture throughout the experiment; ii) donor and target cells separated by a transwell to prevent cell-cell contact; and iii) donor and target cells separated 15 hours after coculture. Peak RT concentration was measured in culture supernatants during a 7-day period after virus inoculation.
